# Protein Hydrolysate from *Pterygoplichthys disjunctivus*, Armoured Catfish, with High Antioxidant Activity

**DOI:** 10.3390/molecules24081628

**Published:** 2019-04-25

**Authors:** Yuchen Guo, Nicholas Michael, Jorge Fonseca Madrigal, Carlos Sosa Aguirre, Paula Jauregi

**Affiliations:** 1 Department of Food and Nutritional Sciences, University of Reading, Whiteknights, Harry Nursten Building, Reading RG6 6AP, UK; gyc19901117@googlemail.com; 2 Centre for Analytical Facilities, Harborne Building, University of Reading, Reading RG6 6UR, UK; n.michael@reading.ac.uk; 3 Universidad Michoacana de San Nicolas Hidalgo, Michoacan, Morelia 58030, Mexico; jfonseca@umich.mx (J.F.M.); csosa@biofermich.com (C.S.A.)

**Keywords:** armoured catfish, enzyme hydrolysate, ABTS, ORAC, FRAP, ACE inhibition, digestibility, peptide sequences

## Abstract

*Pterygoplichthys disjunctivus*, locally named the armoured catfish, is a by-catch of tilapia fishing that accounts for up to 80% of total captured fish in the Adolfo Lopez Mateos dam, in Michoacán, México, affecting the economy of its surrounding communities. This invasive fish is discarded by fishermen since native people do not consume it, partly due to its appearance, yet it is rich in protein. The aim of this study was to produce hydrolysates from armoured catfish using food-grade proteases (neutrases HT and PF and alcalase PAL) and investigate the processing conditions (pH and temperature) that lead to a high degree of hydrolysis, antioxidant activity, and Angiotensin I-Converting Enzyme (ACE) Inhibitory activity. No other similar research has been reported on this underutilized fish. The antioxidant activity was measured by three different methods, ABTS, FRAP and ORAC, with relevance to food and biological systems in order to obtain a more comprehensive assessment of the activity. In addition, the main peptide sequences were identified. All enzymes produced hydrolysates with high antioxidant activity. In particular, the protease HT led to the highest antioxidant activity according to the ABTS (174.68 μmol Trolox equivalent/g fish) and FRAP (7.59 mg ascorbic acid equivalent/g fish) methods and almost the same as PAL according to the ORAC method (51.43 μmol Trolox equivalent/g fish). Moreover, maximum activity was obtained at mild pH and temperature (7.5; 50 °C). Interestingly, the ORAC values obtained here were higher than others previously reported for fish hydrolysates and similar to those reported for fruits such as blueberries, apples and oranges. The peptide sequence IEE(E) was present in several peptides in both hydrolysates; this sequence may be partly responsible for the high antioxidant activity, particularly the one based on iron-reducing power. These findings will be relevant to the valorization of other fish/fish muscle discards and could contribute to the production of food supplements and nutraceuticals.

## 1. Introduction

The loricariid catfish (*Pterygoplichthys disjunctivus*), a species originally from South America [1], is a commercially valuable ornamental fish all over the world, commonly used to clean fish tanks of algae. However, the species has invaded different freshwater bodies throughout the world. In the Adolfo Lopez Mateos dam in Michoacán, México, this invasive fish has found an appropriate habitat in which a lack of predators and exploitation, a surplus of available food and the presence of parental care, as well as a long reproductive season, have produced high recruitment and successful colonization. Locally known as the “bagre armado” or “armoured catfish,” it is a by-catch of tilapia fishing and accounts for up to 80% of total captured fish, thereby affecting the economy of the surrounding communities, which also depend on native species like the Balsas catfish (*Ictalurus balsanus*) and the redside cichlid (*Cichlasoma istlanum*). This invasive fish is discarded by fishermen since native people do not consume it, partly due to its appearance. Fishermen commonly discard these fish directly into the water and at landing areas, generating contamination. However, this fish has a high nutritional value including a high percentage of protein in fish muscle (90.1% of dry weight) and a rich lipid content (fatty acid composition with 39.81% polyunsaturated fatty acids, including 13.9% DHA) [2]. Therefore, the utilisation of these fish as a source of food ingredients with added value can result in an improvement of the local economy. A recent study on zebra fish fed with carp by-product demonstrated a reduction in lipid peroxidation in the muscle and brain, which shows the potential of these fish by-products as nutraceuticals [3].

One option is applying an enzymatic hydrolysis for the solubilization of the protein and a further separation step in order to enhance its nutritional and functional value [4]. The insoluble protein can be used as animal feed, whereas the soluble protein can be used as a source of food ingredients of high commercial value or as a nutrient for bacterial fermentation [5,6]. The main proteases involved in the fish hydrolysis of fish protein are papain, pepsin, neutrase, alcalase, protamex, trypsin and pronase [7,8,9]. Several factors are considered as key to control this complex enzymatic hydrolysis, including the types and concentrations of proteases, pH and temperature of hydrolysis and the source of protein [10]. Typically fish protein hydrolysis is carried out at neutral or alkaline pH conditions as acidic conditions generally lead to lower yields and deterioration of amino acids, with a subsequent reduction in nutritional quality.

The aim of this study was to produce hydrolysates from armoured catfish using food-grade proteases (neutrases and alcalases) and investigate process conditions (pH and temperature) that led to a high degree of hydrolysis, antioxidant activity, and Angiotensin I-Converting Enzyme (ACE) Inhibitory activity. The antioxidant activity was measured by three different methods (ABTS, FRAP and ORAC) based on different mechanisms with relevance to food and biological systems in order to obtain a more comprehensive assessment of the activity. In addition, the main peptide structures in the hydrolysates were determined in order to gain insight into the structure–activity relationship.

## 2. Results

### 2.1. The Degree of Hydrolysis (DH%)

**Figure 1:**
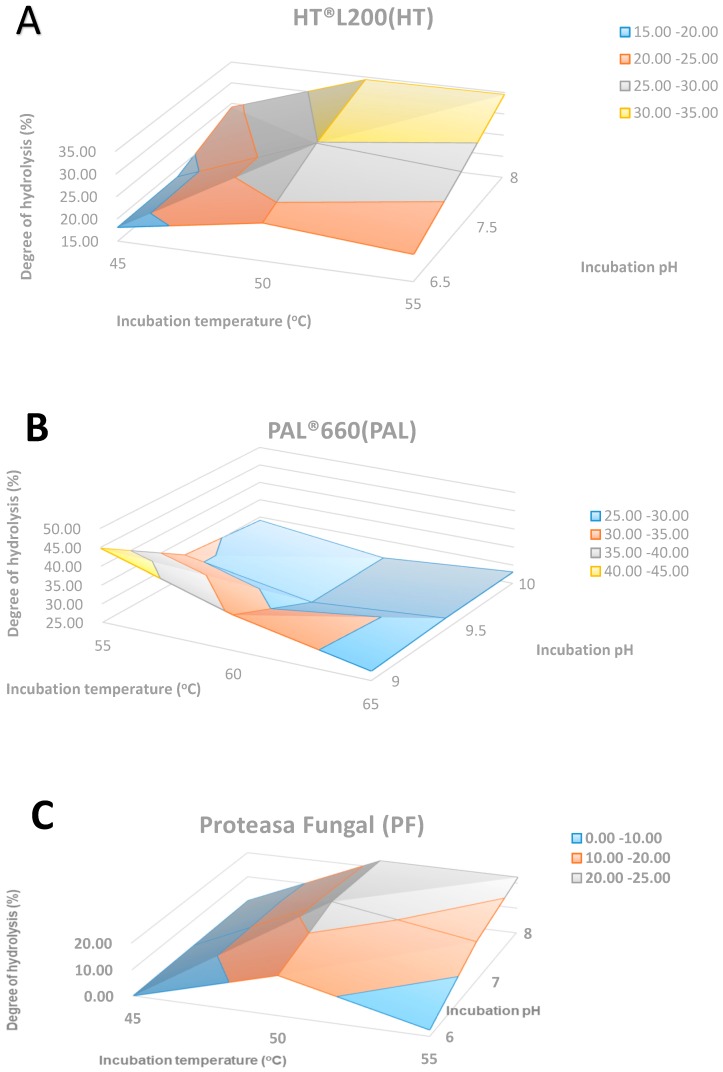
The degree of hydrolysis for each of the enzymes at various pH values and temperatures of hydrolysis: (**A**) HT hydrolysate; (**B**) PAL hydrolysate; (**C**) PF hydrolysate.

The degree of hydrolysis was determined in order to determine the proteolytic activity of the three ENMEX^®^ proteolytic enzymes at different temperatures and pH values (Figure 1A–C) after 2 h of hydrolysis. For HT, the degree of hydrolysis increased with pH and the highest degree of hydrolysis was 34.51% at pH 8 and 55 °C. For PAL the highest degree of hydrolysis was 44.70% at pH 9.0 and 55 °C and the degree of hydrolysis decreased with an increase in pH and temperature. For PF, the maximum degree of hydrolysis was 24.00%, which was achieved at pH 7 and 50 °C. The degree of hydrolysis increased at an alkaline pH. Therefore, the highest degree of hydrolysis was obtained with PAL. However, with this enzyme no maximum was determined as the value of DH% still increased at the highest pH tested (pH 9). To conclude, the degree of hydrolysis was influenced by the type of enzyme, pH and temperature. The values obtained here were higher than those reported for muscle fish of different species—for example, 19.3% degree of hydrolysis in tuna dark muscle hydrolysate with Alcalase and Neutrase [11], 15% degree of hydrolysis in hydrolysate from yellow stripe trevally meat using Alcalase and Flavourzyme [12] and 40% degree of hydrolysis in hydrolysate of the muscle of brownstripe red snapper produced by Alcalase or Flavourzyme [13].

### 2.2. The Antioxidant Activity of Hydrolysates

After producing the hydrolysates of *Pterygoplichthys*, the bioactivity of these hydrolysates was assessed in order to explore the potential health benefits and/or value enhancement of this product. In this study, the antioxidant activity of *Pterygoplichthys* hydrolysates was measured by three different methods, ABTS, FRAP and ORAC. These methods have been widely used in the assessment of antioxidant activity in food and biological systems; they are based on different mechanisms of reaction and act on different free radicals. ORAC measures the antioxidant inhibition of peroxyl radical-induced oxidations and, thus, reflects classical radical chain breaking antioxidant activity by H atom transfer [14]. The ferric reducing antioxidant power (FRAP) measures the antioxidant power based on the reduction of Fe^3+^ (complex ferric ion-TPTZ (2,4,6-tri(2-pyridyl)-1,3,5-triazine)) by the antioxidant [15]. This assay has been used to determine the reducing power in plasma and is a reasonable screen for the ability to maintain redox status in cells or tissues [14]; it is also widely used to assess antioxidant activity in foods as it can be relevant to metal-mediated oxidation of foods; for instance, it has been found to be a good test to asses oxidative deterioration in meat [16]. ABTS has been widely applied to assess antioxidant activity in beverages and foods. In particular, the ORAC and FRAP methods are the most relevant to antioxidant activity in vivo.

#### 2.2.1. The Antioxidant Activity of Hydrolysates by the ABTS Method

**Figure 2:**
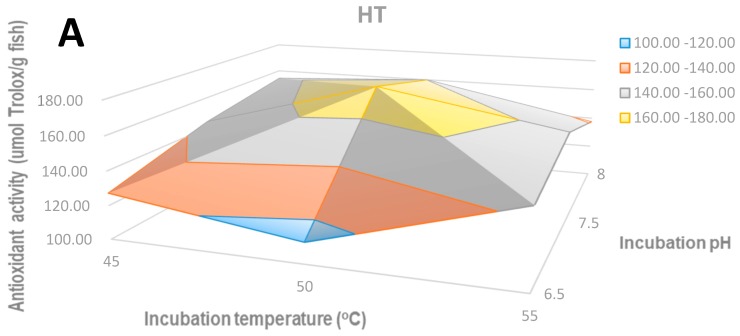

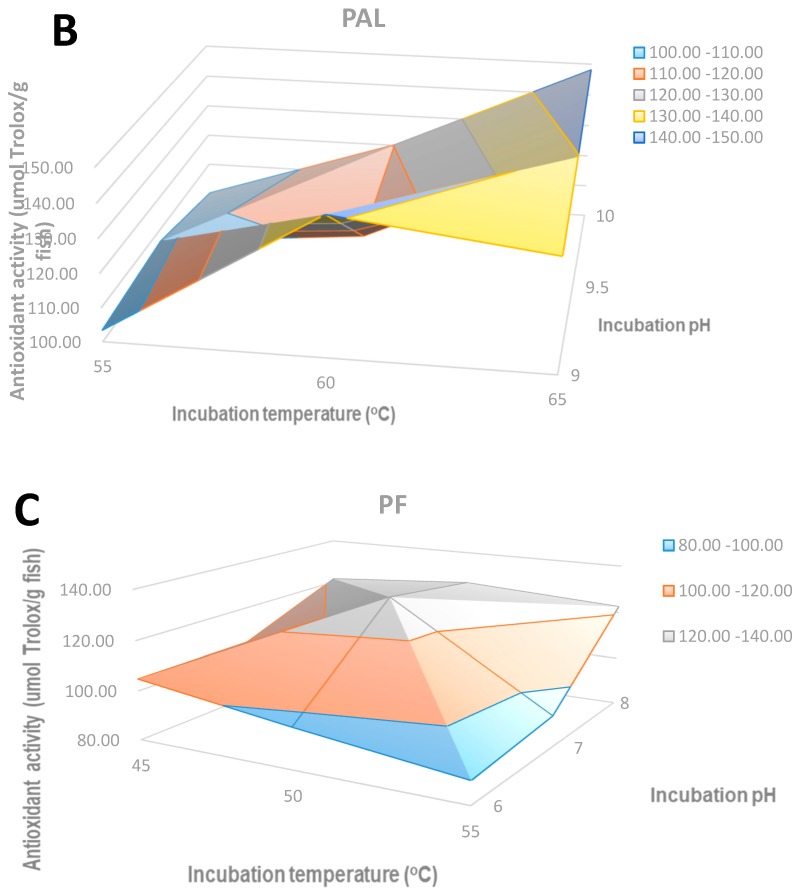
The antioxidant activity of hydrolysates by the ABTS method: (**A**) HT hydrolysate; (**B**) PAL hydrolysate; (**C**) PF hydrolysate.

Figure 2 shows the antioxidant activity of hydrolysates measured as their capacity for scavenging the ABTS radical. The antioxidant activity of HT hydrolysates reached the maximum value at 174.68 μmol/g (Trolox equivalent per gram of fish) (Figure 2A). The antioxidant activity increased from pH 6.5 to 7 but decreased at 8. The pH had a stronger effect than the temperature on the antioxidant activity. Briefly, the antioxidant activity increased slightly when the temperature increased but reduced after 50 °C. The antioxidant activity of PAL hydrolysates reached a maximum at 148.14 μmol/g (Figure 2B) and PF hydrolysates at 131.80 μmol/g (Figure 2C). The hydrolysates of HT and PF reached the highest antioxidant activity at the same conditions that led to the highest degree of hydrolysis, 50 °C/pH 7.5 and 50 °C/pH 7, respectively, which were mild conditions. However, for PAL hydrolysates the highest antioxidant activity was obtained at more extreme conditions, 65 °C and pH 10.

#### 2.2.2. The Antioxidant Activity of Hydrolysates by the FRAP Method

**Figure 3:**
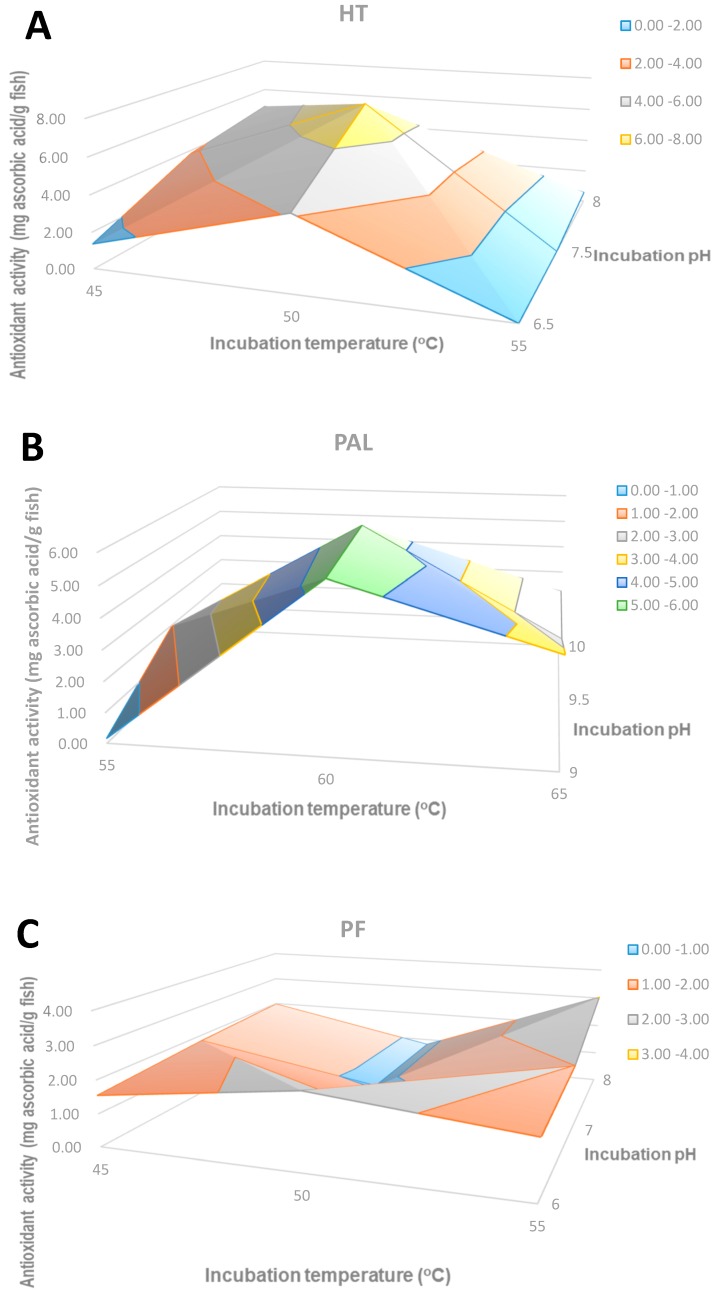
The antioxidant activity of hydrolysates by the FRAP method: (**A**) HT hydrolysates; (**B**) PAL hydrolysates; (**C**) PF hydrolysates.

The FRAP method for antioxidant activity relies on the reduction of Fe^3+^ by the antioxidant and the mechanism of action is based on electron transfer [14]. The antioxidant activity of hydrolysates obtained by the three commercial enzymes at a range of temperatures and pH values was measured and expressed as the ascorbic acid equivalent. The highest antioxidant activity was obtained with HT, where the highest antioxidant activity (7.59 mg Ascorbic acid equivalent per gram of fish) was obtained at 50 °C and pH 7.5 (Figure 3). This peak value was obtained in the middle range of both temperature and pH. On the contrary, for the PF hydrolysates the highest value of antioxidant activity (3.03 mg/g) was obtained at the highest temperature (55 °C) and pH (8). For PAL hydrolysates the maximum activity (5.82 mg/g) was obtained at 60 °C and pH 9.5.

#### 2.2.3. The Antioxidant Activity of Hydrolysates by the ORAC Method

**Figure 4:**
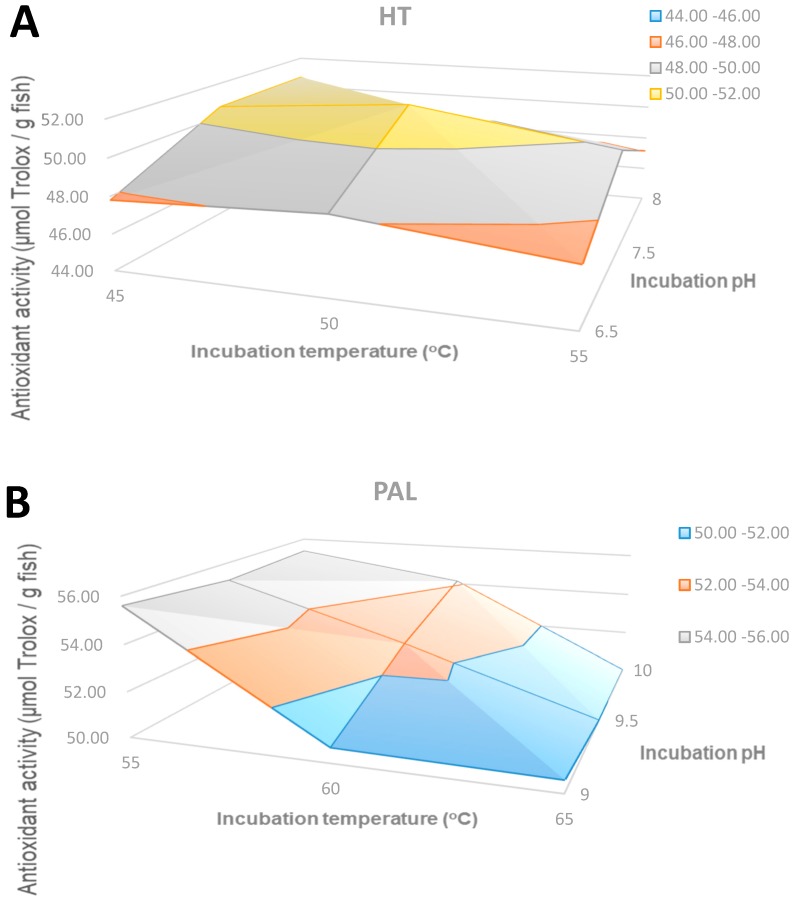

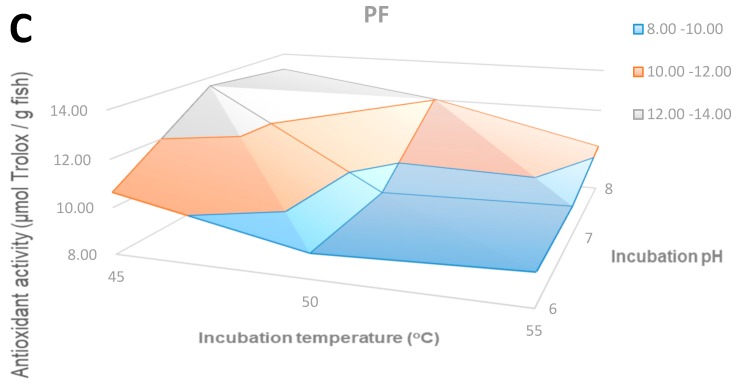
The antioxidant activity of hydrolysates by the ORAC method: (**A**) HT hydrolysate; (**B**) PAL hydrolysate; (**C**) PF hydrolysate.

This method has been applied for the measurement of antioxidant activity in food, especially beverages [15]. As shown in Figure 4A, the optimum conditions for antioxidant activity by the ORAC method with HT were pH 7.5 and 50 °C. The maximum value was 51.43 μmol/g, expressed as Trolox equivalent per gram fish. The antioxidant activity reduced as the temperature increased. Similarly, the antioxidant activity of the hydrolysates by PAL (Figure 4B) reduced as the pH and temperature were raised, and the highest value was 55.60 μmol/g at pH 9 and 55 °C. A similar phenomenon occurred in the PF hydrolysates (Figure 4C) as the highest antioxidant activity (13.60 μmol/g) was at pH 7 and 45 °C, although it was much lower than that in the HT and PF hydrolysates.

### 2.3. The Angiotensin-I-Converting Enzyme (ACE) Inhibitory Activity of Fish Hydrolysates

The ACE inhibitory activity of bioactive peptides from *Pterygoplichthys* hydrolysates produced by three commercial proteolytic enzymes was determined by using FAPGG as the substrate; only the hydrolysate with the highest degree of hydrolysis obtained with each enzyme was assessed. The IC_50_, defined as the concentration of protein/peptide required to reduce the ACE activity by half, was reported as a measure of the effectiveness of the ACE inhibitor (hydrolysate). The IC_50_ values were: 11.84 mg/mL (PAL at pH 9.0 and 55 °C); 11.47 mg/mL (HT at pH 8.0 and 50 °C); 9.58 mg/mL (PF at pH 7.0 and 50 °C).

### 2.4. The Digestibility of the Hydrolysates

The digestibility of the hydrolysates with the highest degree of hydrolysis was measured by the TCA method. The digestibility of the hydrolysates by HT, PAL and PF were 15.71% ± 2.10, 15.13% ± 2.38 and 14.95% ± 2.39, respectively.

### 2.5. Bioactive Peptides

In Table 1 the main peptide sequences identified in hydrolysates produced by each of the proteases at conditions that led to the highest DH% are shown. Only sequences identified with high certainty are included in Table 1, i.e., peptide sequences obtained with a high ion ‘score’ (how well the spectrum matches the suggested peptide) and low ‘expected’ values (the probability of obtaining that peptide purely by chance).

The myofibrillar proteins myosin and actin are the main proteins present in meat (muscle). As expected, the main peptide sequences identified matched these proteins (Table 1); however, often multiple proteins matched the same peptide sequence.

## 3. Discussion

### 3.1. Bioactivity

**Figure 5:**
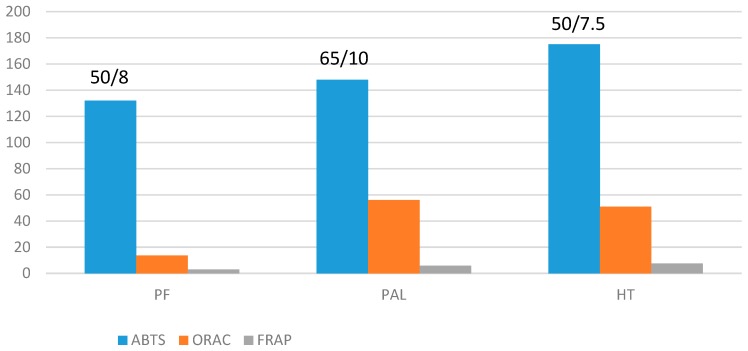
Maximum antioxidant activities obtained for each enzyme according to the three antioxidant activity methods: ABTS (μmol Trolox/g fish), FRAP (mg ascorbic acid/g fish) and ORAC (μmol Trolox/g fish); numbers over ABTS bars indicate T/pH conditions for hydrolysate production.

The hydrolysis of protein by the three enzymes resulted in the generation of peptides with antioxidant activity. PAL produced the hydrolysate with the highest degree of hydrolysis, yet HT produced the hydrolysate with the highest antioxidant activity according to all methods except ORAC (Figure 4); PAL hydrolysate was slightly superior, although similar ORAC results were obtained with HT. These results are in accordance with those reported as the highest degree of hydrolysis was obtained with the alcalase, whilst the highest antioxidant activity was obtained with the neutralase [17,18]. Moreover, with HT maximum antioxidant activity was obtained at the same conditions, pH 7.5 and 50 °C, according to the three methods. The mild conditions in which maximum activity were achieved was another advantage of HT compared to PF and PAL enzymes; this is illustrated in Figure 5, where maximum antioxidant activities for each enzyme and conditions are shown for the ABTS method. Overall, the antioxidant activity based on the ABTS method was superior to that reported for other fish hydrolysates, e.g., 48 μmol/g hydrolysate from the unicorn leatherjacket, DH 40% [19] and was comparable to the value reported for plant-derived extracts, e.g., artichoke extract, 92 μmol/g [20]; blackcurrant extract, 156–196 μmol/g (results from our group, not published); and grape marc extract, 193–485 μmol/g [21].

According to the ORAC method, the highest antioxidant activity was found in the HT (51.43 μmol/g) and PAL (55.60 μmol/g) hydrolysates. These activities were much higher than those reported for hydrolysates from alkaline-aided channel catfish by a *Bacillus* protease (16 μmol/g, which was close to the activity of PF hydrolysates) [22]. Interestingly, the ORAC values obtained here were comparable to those reported for several fruits: in particular, blueberry (48.26 +/−6.49 µmol/g), apple (45.92 +/−2.01 µmol/g), pomegranate (44.79 +/−3.78 µmol/g), orange (28.87 +/−7.17 µmol/g) and red grape (26.05 +/−4.87 µmol of TE/g) [23].

Based on the above results, it was concluded that HT would be the best enzyme to take the process further.

Similar ACE inhibitory activity was obtained with all proteases, and they were close to those reported for hydrolysates from brownstripe red snapper by commercial alcalase (IC50 around 10.0 mg/mL) [13] but lower than that obtained from thornback ray (*Raja clavata*) muscle by Alcalase and Neutrase (around 1.0 mg/mL) [18]. However, that hydrolysate was obtained over a longer hydrolysis time (4 h) than the one produced here (2 h). Also, the hydrolysates obtained here were raw hydrolysates that underwent no further processing; it is known that smaller peptides have higher ACE inhibitory activity and, therefore, further processing by ultrafiltration can render hydrolysates more potent.

### 3.2. Peptide Structure–Activity Relationship

The exact mechanism of antioxidant activity by peptides is not fully understood, yet they have been shown to act as lipid peroxidation inhibitors, scavengers of free radicals and chelators of transition metal ions [24].

Hydrophobic amino acids such as the aliphatic amino acids (Val (V), Leu (L) and Ileu (I)) will enhance the solubility of peptides in lipids and will facilitate access to hydrophobic radicals and hydrophobic PUFAs [24]. Also, aromatic amino acids (His (H) or Tyr (Y)) can donate protons to electron-deficient radicals, which results in them having radical scavenging properties. Acidic and basic groups in the side chain of amino acids (Asp (D), Glu (E), Hys (H), Arg (R), Lys (K)) can act as metal chelators and H donors.

The highest antioxidant activity was found in the hydrolysate by HT (Figure 5). It is interesting to note that most of the peptides identified in this hydrolysate (Table 1) contain several acidic amino acids in their sequences: in particular, glutamic acid (E) and aliphatic amino acids (Ala(A), Ileu (I), Leu (L)). These sequences were found in both PAL and HT hydrolysates, which had the highest FRAP activity. In particular, the sequence IEE(E) is repeated in several peptides in both hydrolysates (Table 1). This combination of aliphatic and acid amino acids was also found in the peptide LEELEEELEGCE from frog skin, which showed high antioxidant activity [17]. According to these authors, the IC50 (concentration of peptide at 50% inhibition) for the purified peptide against a range of free radicals was from 12.8 to 32.6 μM; this would be equivalent to about 1000 μM Trolox for 50% inhibition against ABTS radical. It is expected that acidic amino acids would be effective at iron reduction (as measured by FRAP), in a similar manner to ascorbic acid [25]

On the other hand, the peptides in the PF hydrolysate contain more aromatic amino acids than the peptides in the other two hydrolysates and some peptides rich in hystidine (H). This hydrolysate had the lowest FRAP activity, which correlates well with the lower presence of acidic amino acids in the peptide sequences as compared to HT and PAL hydrolysates.

## 4. Materials and Methods

### 4.1. Materials

The *Pterygoplicgthys* samples are supplied by the University of Michoacan of San Nicolas de Hidalgo (Morelia, Mexico). The enzymes were supplied by ENMEX (Tlalnepantla de Baz, Mexico): two neutrases, HT Proteolitic ^®^ L200 (HT), and Proteasa Fungal (PF) and one alcalase, PAL^®^660 (PAL). The following chemicals were purchased from Sigma Aldrich^®^ (Gillingham, UK): Bis-tris Propane (B6755); O-phthaldialdehyde (OPA, P1378); Sodium-dodecyl-sulphate (SDS) (L3771); Dithiothreitol 99% (DTT, D0632); Potassium persulfate (216224) was purchased from Sigma Aldrich^®^; 2,2′-Azino-bis(3-ethylbenzothiazoline-6-sulfonic acid) diammonium salt (ABTS, A1888); 2,4,6-Tris(2-pyridyl)-s-triazine (TPTZ, T1253); Ferric Chloride Hexahydrate (F2877); Sodium Acetate Trihydrate (S8625); Glacial Acetic Acid (320099); Disodium fluorescein (F6377); 2,2′-Azobis(2-methylpropionamidine) dihydrochloride (AAPH, 440914); Sodium chloride [NaCl] > 99.5% (S7653); Tris (hydroxymethyl) methylamine (T1503); Angiotensin-converting enzyme (ACE, A6778; Hydrochloric acid 36.5–38.0%, (H1758); FAPGG—*N*-[3-(2-furyl)acryloyl]-Phe-Gly-Gly ≥98% (F7131); Glycerol solution 86–89% (49781); Trichloroacetic acid (TCA, T6399).

### 4.2. Methods

#### 4.2.1. The Preparation and Pre-Treatment of Fish Protein Hydrolysis

**Figure 6:**
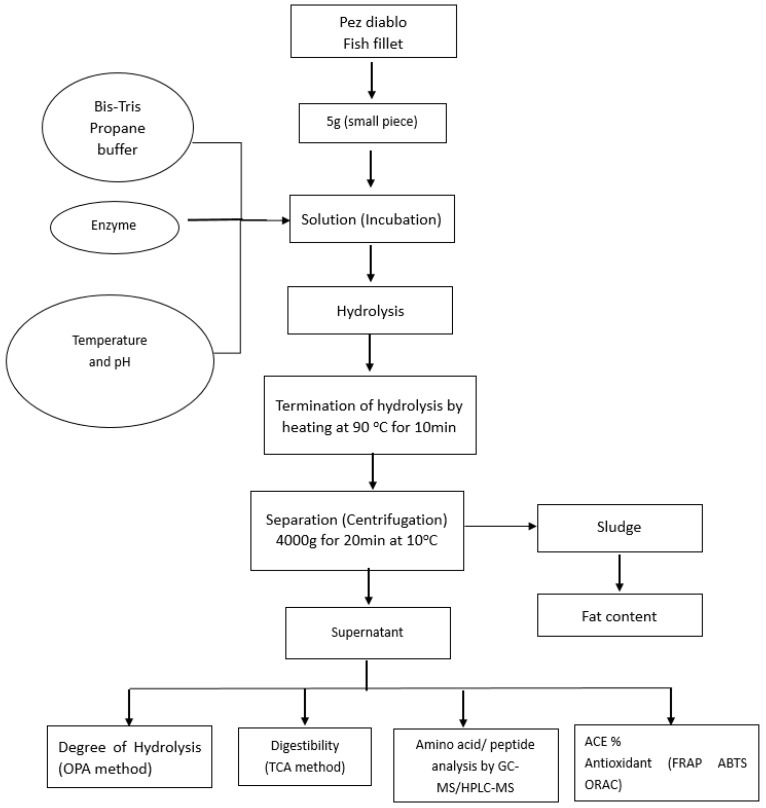
Process followed for the preparation of *Pterygoplichthys disjunctivus* hydrolysates.

The preparation of armoured catfish (*Pterygoplichthys disjunctivus*) hydrolysates is described in Figure 6. Five grams of fish fillet were cut into small pieces and added into 50 mL 0.1M Bis-tris Propane buffer (pH 11). Then the pH of each sample was adjusted to the target pH as described in Table 2. The 0.2% *w*/*v* (*v*/*v*) enzyme was added into the sample and the incubation started at a specific temperature for 120 min. Every 30 min, the pH of the sample was recorded and adjusted back to the starting pH. At the end of hydrolysis, the pH was measured and hydrolysis was ended by immersing the sample in a 90 °C water bath for 10 min to inactivate the enzyme (note: the temperature of the sample reached 85 °C). The pH of the sample was changed to pH 7. The sample was cooled down in an ice bath to room temperature and centrifuged at 4000 g at 10 °C for 20 min in a Thermo Multifuse 3SR+ (Thermo Fisher Scientific, Hemel Hempsted, UK). The supernatant was collected in a 5-mL sterile plastic bottle and stored at −20 °C for further analysis.

#### 4.2.2. Experimental Design

A 3^2^ factorial design was applied where the two dependent variables were pH and temperature and each was studied at three levels (−1, 0, 1); the experimental conditions are shown in Table 2. Each experiment was carried out once for each enzyme, but the antioxidant activity was measured in triplicate and the ACEi% in duplicate.

#### 4.2.3. Determination of the Degree of Hydrolysis

The degree of hydrolysis was determined by applying the o-phthaldialdehyde (OPA) method [26] with some modifications. The concentration of samples was kept between 250 and 500 μg mL^−1^. To prepare 200 mL of solution, 7.620 g of di-sodium tetraborate decahydrate and 200 mg of sodium-dodecyl-sulfate (SDS) were dissolved completely in 150 mL of water until homogenised in a flask of 250 mL. Separately, 160 mg of *O*-Phthaldialdehyde (OPA) were dissolved in 4 mL of ethanol in a 10-mL flask then transferred to the solution mentioned above, rinsing the small flask completely with deionized water. Then 176 mg of Dithiothreitol 99.0% (DTT) were added to the solution and stirred; this was transferred into a 200-mL volumetric flask and filled up to 200 mL with deionized water. Standard or samples (200 μL) were added into 1.5 mL OPA reagent in 2-mL acryl cuvettes and after 2 min incubation the absorbance was measured by a UV-Vis Spectrophotometer (Ultrospec ^®^ 1100 pro, GE Healthcare, Amersham, UK) at 340 nm.

Deionised water was used as a blank and a serine dilution was used as standard. The DH was calculated using the following equation:

(1) $$DH\%=\frac{h}{h_{tot}}\times 100\%,$$

where *DH*% is the degree of hydrolysis in percentage, *h* ($meqv\;g^{-1}$) is the number of hydrolysed bonds and *h_tot_* is the total number of peptide bonds per protein equivalent; *h_tot_* is dependent on the protein source and for fish, *h_tot_* is 8.6 $meqv\;g^{-1}$. The value of h was obtained by applying the equations below:

(2) $$h=\frac{\left(Serine\cdot NH_{2}-\;\beta \right)}{\left(\alpha \right)}$$

(3) $$Serine\cdot NH_{2}=\frac{\left(OD_{sample}-OD_{blank}\right)}{\left(OD_{standard}-OD_{blank}\right)}*0.9516\;meqv\;L^{-1}\times \frac{D}{P}$$

where serine-NH_2_ = meqv serine NH_2_ g^−1^ protein; D = dilution factor; P = protein concentration in sample (g L^−1^).

#### 4.2.4. Determination of Antioxidant Activity of Hydrolysates

##### The ABTS Free Radical Scavenging Activity Assay

The total antioxidant activity of samples was measured by ABTS assay at 734 nm, with some modifications from that reported [27]. The ABTS^•+^ stock solution was prepared by mixing 5 mL ABTS solution (7 mM) and 88 μL potassium persulfate (140 mM K_2_S_2_O_8_) solution together. Then, the mixture was stored in the dark at room temperature for at least 16 h prior to use. The working solution of the ABTS^•+^ was obtained by diluting the ABTS^•+^ stock solution with phosphate-buffered saline (PBS pH 7.4) to an absorbance of 0.70 ± 0.02 at 734 nm. Twenty microliters of samples were added into 2 mL ABTS^•+^ working solution, and the solution was homogenised by 1 min vortex. The solution was then incubated in the dark for 6 min, and the absorbance (ABS_sample/standard_) was recorded at 734 nm using a UV-Vis Spectrophotometer (Ultrospec ^®^ 1100 pro). The absorbance of the ABTS^•+^ working solution was measured at the same wavelength and used as a control (ABS_control_). The PBS was used as a blank. The (scavenging) activity was measured based on the difference between ABS_control_ and ABS_sample_. A standard curve of trolox (0–2000 μM) against scavenging activity was constructed in order to relate scavenging activity to Trolox equivalents. The antioxidant activity was expressed as µmol Trolox equivalent/g fish.

##### The Ferric Reducing Antioxidant Power (FRAP) Assay

The total antioxidant activity of samples was determined through ferric reducing antioxidant power (FRAP) [28]. The stock solution of FRAP method included: 25 mL acetate buffer (300 mM), 2.5 mL 2,4,6-tripyridyl-s-triazine (TPTZ) solution (10 mM in 40 mM HCl), and 2.5 mL ferric chloride hexahydrate aqueous solution (20 mM). Then, 10 μL of the sample/standard were added into 300 μL FRAP reagent in a microcentrifuge tube and vortexed for 10 s. Then 100 μL of this mixture, in triplicate, were transferred into the microwell plate (96-well, NUNC, FB) and absorbance was measured at 595 nm by a computer-controlled Tecan Microplate reader (Tecan Ltd., Reading, UK). Results were expressed as ascorbic acid equivalent (AAE) using an ascorbic acid (0.001761 mg/mL–0.1761 mg/mL) standard curve (The absorbance=1.8877×AA concentration−0.0013, *R*^2^ = 0.9923).

##### The Oxygen-Radical Absorbing Capacity (ORAC) Assay

The total antioxidant activity of samples was also measured by oxygen-radical absorbing capacity (ORAC) method, based on previously reported methods [29,30]. A 96-well plate and a Tecan Genius plate reader were used for the fluorescence measurements. The emission and excitation wavelengths were set to 535 and 485 nm, respectively, at 37 °C. Twenty-five microliters of the samples were mixed with 150 μL disodium fluorescein (96 nM in phosphate buffer pH 7.4). Then, a volume of 75 μL 2,2′-Azobis(2-amidinopropane) dihydrochloride (AAPH, 153 Mm kept in ice) was added to initiate the oxidation reaction. The kinetic fluorescence reading of the samples was recorded for 30 cycles with a 60 s per cycle setting. The antioxidant capacity was expressed as the area under the curve (AUC) by applying Equation (4) below:

(4) AUC=1+RFU1/RFU0+RFU2/RFU0+RFU3/RFU0+…+RFUn/RFU0

where RFU_0_ = relative fluorescence units at time point zero; RFU_n_ = relative fluorescence units at time points;

A Trolox standard solution and subsequent dilutions (3.125–50 μM) were prepared to construct a calibration curve and PBS buffer was used as a blank. The antioxidant capacity of the sample was expressed as Trolox equivalents (μmol Trolox/g fish).

#### 4.2.5. Determination of the Angiotensin-I-Converting Enzyme (ACE) Inhibitory Activity of Fish Hydrolysates

The ACE inhibitory activity (ACEi%) of the fish protein hydrolysates was determined according to the methods reported [31,32] and with some modifications. The tripeptide *N*-[3-(2-furyl) acryloyl]-Lphenylalanyl-glycyl-glycine (FAPGG) was used as the substrate of the interaction with ACE in a 96-well microplate at 37 °C. Briefly, 150 μL of 88 mM FAPGG in Tris-HCl (50 mM, pH 7.5 and 300 mM NaCl) buffer were mixed with 10 μL of ACE enzyme (0.25 mU in 50% Tris-HCl buffer and 50% glycerol) and 10 μL of the sample. The kinetics of absorbance of the mixture was monitored at wavelength 340 nm for 30 min at 1-min interval by the Tecan plate reader. The slope of decreasing absorbance of the samples was the indicator of the enzyme activity; therefore, the inhibitory activity of each hydrolysate was calculated by Equation (5):

(5) $$ACE\;Inhibitory\;\left(\%\right)=\left(1-\frac{\rho_{i}}{\rho_{0}}\right)$$

where *ρ_i_* is the slope in the presence of hydrolysate (inhibitor) and *ρ*_0_ is the slope with deionized water. Also, in order to compare different inhibitors (hydrolysates), the IC_50_ value, which is defined as the protein concentration required to inhibit 50% of the ACE enzyme activity, was determined.

#### 4.2.6. The Digestibility of the Hydrolysates

The in vitro digestibility of the sample was determined by previously reported methods [33,34]. The hydrolysate was mixed with 20% trichloroacetic in the ratio of 50:50 and then incubated at room temperature for 30 min. After incubation, the sample was centrifuged for 10 min at 4 °C and 3000 *g*. The supernatant was collected and the soluble protein concentration was determined by bicinchoninic acid assay. The digestibility of the hydrolysate was expressed as the percentage of soluble protein in relation to total protein.

Total protein in fish was measured as 19.8% wet weight. Protein determination was carried out through the evaluation of the total nitrogen using a Scorpio Scientific Kjeldahl unit (Neocitec, Mexico City, Mexico) and following the certified method NMX-F-608-NORMEX-2011. The conversation factor was 6.25. The sample (1 g) was subjected to acid digestion using sulfuric acid; the product was taken to an automatic distiller. The distilled sample was titrated with 0.1 N hydrochloric acid.

#### 4.2.7. Identification of Peptides

Hydrolysates produced by each of the enzymes at the conditions that led to maximum hydrolysis were chosen for further analysis to identify the main peptide sequences.

Samples (10 μL) were injected on a Thermo Scientific Accela HPLC system interfaced to a Thermo Scientific LTQ Orbitrap XL mass spectrometer. The column was a Thermo Scientific Hypersil Gold C18 50 × 2.1 mm with particles of 1.9 microns in size and pores of 175 Å. Mobile Phase A was water and Mobile Phase B was acetonitrile; both contained 0.1% formic acid. The gradient was 0–2 min held on 5%B; 2–20 min 5–20%B; 20.1–23 min held on 80%B; 23.1–30 min held on 5%B. An electrospray ionisation (ESI) source operating in positive ion mode was used. The salient source settings were: Capillary temperature 300 °C, Sheath and Aux nitrogen gas flow 45 and 10 arbitrary units, respectively. Source voltage; 4 kV, Capillary voltage; 31, Tube Lens; 131. The instrument was operating a data-dependent acquisition (DDA). Scan event 1 was acquiring full-scan MS over the *m*/*z* range 400–2000, at resolution 30,000 in the Orbitrap. Scan event 2 was acquiring LTQ ion trap Collision-Induced Disassociation (CID) of significant multiply charged peaks found in scan event 1, which were scanned out in the LTQ ion trap.

Mascot Generic Format (MGF) files were generated from the Thermo Raw files using ProteoWizard 3.0.11148 32 bit and these were searched using an in-house Mascot server v2.5.0 (Matrix Science Ltd., London, UK). Search parameters were: peptide mass 10 ppm, fragment mass 0.6 Da; No enzyme; Variable modifications: Acetyl (Protein *N*-term),Gln->pyro-Glu (*N*-term Q),Oxidation (M); Database: NCBInr 20160712; Taxonomy: Chordata (vertebrates and relatives). Reports were formatted with the “expected cutoff” set to 0.05.

#### 4.2.8. Statistical Analysis

All the measurements were carried out at least in duplicate. The analysis of variance was done by XLSTAT v20.1 for comparison among samples with different treatments (i.e., different enzymes and/or the same enzyme but different pH and temperature conditions). The confidence level was set to *p* ≤ 0.05.

## 5. Conclusions

For the first time, the production of a protein hydrolysate from armoured catfish (*Pterygoplichthys disjunctivus*) by a range of proteases has been investigated. High antioxidant activity was obtained in the hydrolysates produced by the three enzymes. In particular, HT led to the highest antioxidant activity according to the ABTS and FRAP methods and almost the same as PAL according to the ORAC method. Moreover, a further advantage of using HT was that the best results were obtained under mild temperature and pH conditions. Interestingly, the ORAC values obtained here were higher than others previously reported for fish hydrolysates and similar to those reported for fruits such as blueberries, apples and oranges. Moreover, both PAL and HT hydrolysates contained peptide sequences rich in glutamic acid and aliphatic amino acids such as alanine, leucine and isoleucine. In particular, the sequence IEE(E) was present in several peptides in both hydrolysates; this sequence may be partly responsible for the high antioxidant activity, particularly for the activity based on the iron reducing power (FRAP method). Overall, these results show that this underused fish is an important source of antioxidant peptides that can be developed further as food supplements and/or natural antioxidants in food formulations.

## Figures and Tables

**Table 1:** Main peptide sequences identified in each of the enzyme’s hydrolysates; Mr(expt) and Mr(calc) are the experimental and calculated theoretical molecular weights; ‘Protein’ is the protein matching that peptide.

Enzyme	Peptide	Mr(expt)	Mr(calc)	Score	Expect	Protein
**PAL**	REELNEIIEEVDEDGSGT	2032.9123	2032.9073	100	2.7 × 10^−5^	Troponin C
	REELNEIIEEVDEDGSGTID	2261.0018	2261.0183	85	7.3 × 10^−4^	
	IAEKDEEIEQLK	1443.7383	1443.7456	79	4.9 × 10^−3^	Embryonic myosin heavy chain
	IEELEEEIEAER	1487.6950	1487.6991	88	4.8 × 10^−4^	
	KKAEPAPAPAPAPE	1372.7291	1372.7350	76	0.009	Embryonic myosin light chain
**HT**	LAEKDEEIEQI	1315.64	1315.6507	71	0.03	Myosin heavy chain
	LAEKDEEIEQIK	1443.7384	1443.7456	78	0.0056	
	IEELEEEIEAER	1487.6917	1487.6991	90	2.5 × 10^−4^	
	NSYEEALDHLETL	1532.6970	1532.6994	68	0.045	
	MDLENDKQQSEEK	1592.6925	1592.6988	68	0.03	
	IMDLENDKQQSEEK	1705.7735	1705.7828	80	0.0028	
	TERLEDEEEINAE	1575.6856 1575.6900		71	0.03	
	LESEVEGEQR	1174.5428 1174.5466		70	0.03	
	FDMFDTDGGGDISTK	1604.6567 1604.6665		109	9.5 × 10^−7^	
	REELNEIIEEVDEDGSGTID	2261.0113 2261.0183		73	0.012	
	SQKEDKYEEEI	1396.6302 1396.6358		82	0.015	
	LEKTIDDLEDELYSQ	1809.8489 1809.8520		98	6.2 × 10^−5^	
	GQKDSYVGDEAQSK	1510.6838	150.6900	97	2.3 × 10^−5^	Mutant beta actin (*homo sapiens*)
	SIIDQDKSGFIEEDELKL	2078.0318	2078.0419	91	4 × 10^−4^	Parvalbumin
	GDTDGDGKIGVDEF	1423.6112	1423.6104	88	2.2 × 10^−4^	Parvalbumin beta
	KQFLEELLTTQ	1348.7228	1348.7238	74	0.012	Myosin light chain 2
	IVGDDLTVTNPK	1270.6723	1270.6769	74	0.011	Enolase
**PF**	DDLQAEEDKVNT	1375.6122	1375.6103	71	0.016	Myosin heavy chain fast skeletal muscle-like [*Ictalurus punctatus*]
	TEEMASQDESIAK	1437.6256	1437.6293	97	3.3 × 10^−5^	
	AQRLQEAEESIEAV	1571.7776	1571.7791	71	0.029	
	QGEVEDLMIDVERA	1602.7473	1602.7559	86	7 × 10^−4^	
	RNAEEKAKKAITDAA	1614.8767	1614.8689	74	0.012	
	LEEAEGTLEHEESKI	1712.8086	1712.8104	87	7 × 10^−4^	
	EELKKEQDTSAHLER	1811.8991	1811.9013	80	0.0047	
	LEEAEGTLEHEESKIL	1825.8829	1825.8945	72	0.026	
	KRQAEEAEEQANTHLS	1839.8700	1839.8711	71	0.026	
	REQFEEEQEAKAELQ	1862.8581	1862.8646	86	7.6 × 10^−4^	
	EQQVDDLEGSLEQEKK	1873.8915	1873.8905	71	0.034	
	AEELKKEQDTSAHLER	1882.9359	1882.9384	92	0.00031	
	QARIEELEEEIEAERAA. + Gln->pyro-Glu (N-term Q)	1967.9345	1967.9435	78	0.0063	
	KQKYEEGQAELEGAQKEA	2034.9739	2034.9857	92	2.4 × 10^−4^	
	EMEEAQERADIAESQVNK	2075.9393	2075.9429	81	0.0021	
	KRENKNLQQEISDLTEQI	2185.1241	2185.1338	72	0.034	
	KLEQQVDDLEGSLEQEKKL	2228.1563	2228.1536	89	0.00059	
	HELEKAKKTVETEKSEIQTA	2298.2000	2298.2067	84	0.002	
	RKVQHEMEEAQERADIAESQVNK	2724.3189	2724.3249	104	2 × 10^−5^	
	EEGQAELEGAQKEARS	1730.8073	1730.8071	86	7.5 × 10^−4^	Myosin heavy chain, fast skeletal muscle isoform X1 [*Danio rerio*]
	KMEIDDLSSNMEAVAKS	1866.8692	1866.8703	86	8.2 × 10^−4^	Myosin heavy chain (*Seriola demirili*)
	SYKRQAEEAEEQANTHLS	2089.9620	2089.9664	72	0.02	Myosin heavy chain-2 [*Thunnus orientalis*]
	AEQELLDASERVGL	1528.7728	1528.7733	71	0.027	Myosin heavy chain, fast skeletal muscle-like [*Clupea harengus*]
	EADLVQIQGEVDDTVQEA	1957.9077	1957.9117	96	8.8 × 10^−5^	Myosin heavy chain [*Pennahia argentata*]
	KAISEELDHALNDMTSI	1885.9049	1885.9091	84	0.0017	Tropomyosin alpha-1 chain-like isoform X2 [*Nothobranchius furzeri*]
	EKTIDDLEDELYSQKLK	2066.0377	2066.0419	82	0.0028	
	KLEKTIDDLEDELYSQKL	2179.1241	2179.1259	87	9.1 × 10^−4^	
	KATEDELDKYSEALKDAQEKL	2423.1937	2423.2067	88	8.4 × 10^−4^	
	RALGQNPTNKDVAK	1510.8211	1510.8216	78	0.0039	Myosin light chain 1 [*Thunnus thynnus*]
	KKAEPAPAPAPAPE	1372.7315	1372.7350	76	0.0087	
	SSSSLEKSYELPDGQVI	1837.8938	1837.8945	71	0.032	Alpha-smooth muscle actin-rabbit (fragment)
	SSSSLEKSYELPDGQVIT	1938.9372	1938.9422	71	0.038	
	AVFDISNADRLGSSEVDQV	2020.9632	2020.9702	82	0.0027	Creatine kinase M-type [*Gekko japonicus*]
	GDFSADQIEDFKEA	1612.6844	1612.6893	76	0.0031	Myosin light chain 1/3, skeletal muscle isoform [*Cynoglossus semilaevis*]

**Table 2:** Conditions for each of the enzymes.

Enzyme	Temperature (°C)			pH			#%(*w*/*v*)	Time (min)
**HT PROTEOLITIC^®^L 200**	45	50	55	6.5	7.5	8.0	0.2%	120
**PAL^®^660**	55	60	65	9.0	9.5	10.0	0.2%	120
**Proteasa Fungal**	45	50	55	6.0	7.0	8.0	0.2%	120

#%*w*/*v* is the enzyme (mass) to buffer solution volume ratio as a percentage.
